# A phase-field model by an Ising machine and its application to the phase-separation structure of a diblock polymer

**DOI:** 10.1038/s41598-022-14735-4

**Published:** 2022-06-24

**Authors:** Katsuhiro Endo, Yoshiki Matsuda, Shu Tanaka, Mayu Muramatsu

**Affiliations:** 1 Graduate School of Science and Technology, Keio University, 3-14-1 Hiyoshi, Kohoku-ku, Yokohama, Kanagawa 223-8522 Japan; 2 Fixstars, 3-1-1 Shibaura, Minato-ku, Tokyo, 108-0023 Japan; 3 Green Computing System Research Organization, Waseda University, 27 Wasedacho, Shinjuku-ku, Tokyo, 162-0042 Japan; 4 Department of Applied Physics and Physico-Informatics, Keio University, 3-14-1 Hiyoshi, Kohoku-ku, Yokohama, Kanagawa 223-8522 Japan; 5 Department of Mechanical Engineering, Keio University, 3-14-1 Hiyoshi, Kohoku-ku, Yokohama, Kanagawa 223-8522 Japan

**Keywords:** Mechanical engineering, Computational methods, Polymers

## Abstract

A novel model to be applied to next-generation accelerators, Ising machines, is formulated on the basis of the phase-field model of the phase-separation structure of a diblock polymer. Recently, Ising machines including quantum annealing machines, attract overwhelming attention as a technology that opens up future possibilities. On the other hand, the phase-field model has demonstrated its high performance in material development, though it takes a long time to achieve equilibrium. Although the convergence time problem might be solved by the next-generation accelerators, no solution has been proposed. In this study, we show the calculation of the phase-separation structure of a diblock polymer as the equilibrium state using phase-field model by an actual Ising machine. The proposed new model brings remarkable acceleration in obtaining the phase-separation structure. Our model can be solved on a large-scale quantum annealing machine. The significant acceleration of the phase-field simulation by the quantum technique pushes the material development to the next stage.

## Introduction

Diblock polymers are a widely used material for structures^1^. The microstructure of a diblock polymer changes depending on the synthetic conditions. The process of the change in microstructure is spinodal decomposition, and various phase-separated structures, such as lamellar, cubic, hexagonal, and gyroid structures, can be obtained^2^. Since this material structure has a strong influence on mechanical properties^3^, it is very important to predict which structure will be obtained experimentally.

To date, several methods have been developed to analyze the equilibrium state of diblock polymers, such as the molecular dynamics^4^ Monte Carlo method^5^, the self-consistent field theory^6^ and the phase-field model^7,8^. The phase-field model is a continuum model that expresses the interface with a smooth function using a variable called the order parameter. This model has the advantage of being free from solving complicated boundary value problems. The governing equation can be derived in a relatively simple form. Hence, it is widely used to reproduce various material structures^1,9–16^. The governing equation of the phase-field model is formulated by variation of the energy functional. However, conventional analysis sometimes takes a very long time to obtain an equilibrium state, which is a problem when a large-scale simulation is required. Hence, various schemes have been proposed to solve this problem^17–19^, which is the key to accelerating material development.

On the other hand, next-generation accelerators including quantum computers are steadily developing. In particular, quantum annealing machines^20–23^ can search for the minimum value of the objective function at high speed. They are often applied to combinational optimization problems involving the objective functions of the target. If the objective function can be replaced with energy expressing the target phenomenon, quantum acceleration of finding of equilibrium state acquisition can be expected by a physics simulation^24–27^.

In this study, we propose an objective function for exploring the phase-separated structure of diblock polymers by annealing, with an eye toward the use of quantum annealing. On the basis of the obtained results, we confirm the tendency of the microstructure and the simulation performance by comparison with the phase-field simulation. The approach is based on the use of a global optimization metaheuristic algorithm, called simulated annealing, to directly minimize the Helmholtz free energy instead of minimizing it analytically and then solving the resulting nonlinear, partial differential equation, i.e., the governing equation of the phase-field model. In this paper, we illustrate the use of simulated annealing in the solution of the phase-field model by applying it to the formation of a microstructure in a diblock polymer.

First, we review the results of the conventional analysis method of the phase-field model and propose the Ising model for solving the phase-field model in the following section. Subsequently, we present the calculation results of the newly defined quantity for the Ising model. Ising machines are used as a dedicated accelerator for solving Ising model, and from the simulation by the Ising machine, we find remarkable results, which indicate the effectiveness of the proposed approach. Finally, we conclude with remarks on the possibilities of a much larger-scale simulation and on the application of the annealing method to other problems related to continuum mechanics.

**Figure 1:**
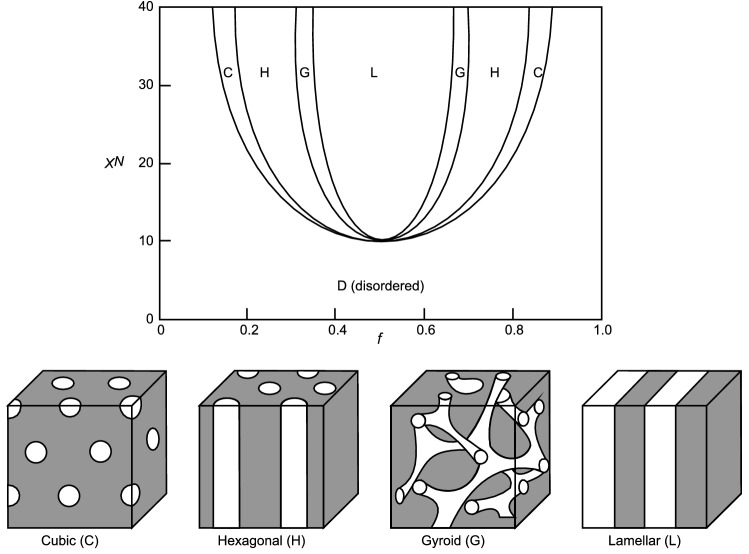
(Top) Phase diagram of a diblock polymer. The horizontal axis is the fraction of a monomer $f$, while the vertical axis shows $\chi N$, the product of the polymerization $N$ and the interaction parameter $\chi$. (Bottom) Schematics of the phases of a diblock polymer.

To verify the problem of spinodal decomposition for diblock polymers, we carry out conventional phase-field simulations. A phase diagram of the phase-separated structure in the diblock polymer is shown in Fig. 1^2^. The phase-field model is composed of the gradient energy, Flory–Huggins interaction energy^28,29^, and Ohta-Kawasaki long-distance interaction energy^30^. The fineness of the phase-separation structure is controlled by the magnitude of the Ohta-Kawasaki energy. The governing equation is derived through the Cahn–Hilliard equation^30^ and discretized by the finite difference method following the conventional manner. The mobility $M$ in the analysis is employed as $M=1.0\, {\mathrm{s}}^{-1}$. The analysis area of $32 \,\upmu\mathrm{m}\times 32\,\upmu\mathrm{m}$ is discretized into $64\times 64$ grids under the periodic boundary condition.

**Figure 2:**
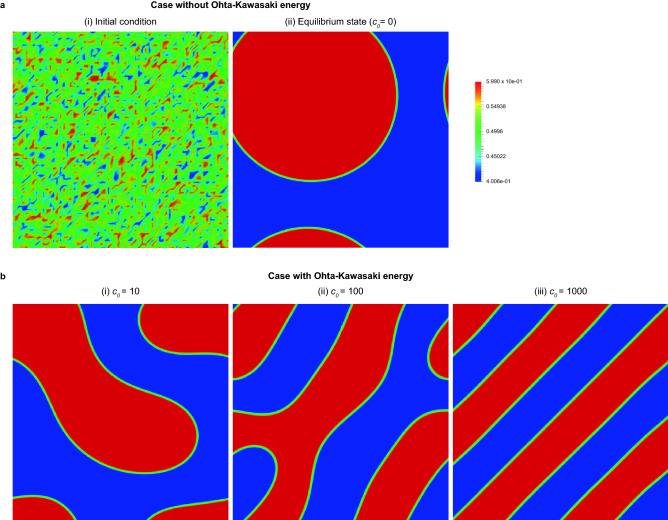
(**a**) Results of the phase-field analysis without considering the Ohta-Kawasaki energy at the (i) initial state and (ii) equilibrium state. A cubic or two-dimensional hexagonal structure is observed at the (ii) equilibrium state. (**b**) Parametric study of the phase-field analysis with the change in strength of the Ohta-Kawasaki energy. The stronger the Ohta-Kawasaki energy becomes, the finer the microstructure at the equilibrium state.

The initial distribution is given by random noise around $f=0.5$, which is shown in Fig. 2a(i). First, we conduct a numerical analysis with the constant of long-distance interaction terms ${c}_{0}$ equal to zero. Figure 2 a(ii) shows the equilibrium state obtained by phase-field analysis. Polymer A is shown in blue, and polymer B is shown in red, being separated into two large phases. These large phases are considered to minimize the surface energy. Next, we change the parameter *c*_0_ in the long-distance term derived from the Ohta-Kawasaki energy^30^. We confirm that the incremental error at the final computational step becomes less than $1.2\times {10}^{-6}$ in all cases. The incremental error is defined as the product of the time increment per step and the maximum increment of the order parameter per step among all the grids. In Fig. 2b, the separation pattern, which is different from that in Fig. 2a, becomes finer with increasing parameter *c*_0_, which is considered to be induced by the effect of the long-distance energy term of the Ohta-Kawasaki energy. Additionally, the phase-separation structure in Fig. 2a(ii) seems cubic or hexagonal, as in Fig. 1, while that in Fig. 2b(iii) is lamellar-like, as in Fig. 1. The effect becomes more significant as the effect of the Ohta-Kawasaki energy increases. Both simulations take approximately 5960 s.

We performed a new phase-field simulation using an Ising machine which solves one of the formalized combinatorial optimization problems, i.e., quadratic unconstrained binary optimization (QUBO). QUBO uses a set of binary states. The objective function, called the Hamiltonian, is composed only of the quadratic polynomials of the states. One of the advantages of Ising machines is that once the problem is well formalized as a QUBO-style Hamiltonian, Ising machines can be used to solve the problem without paying attention to the details of numerical calculations, such as the discretization error of finite time steps and the solver implementation method.

Our proposed method formalizes the problem of spinodal decomposition for diblock polymers into the QUBO-style Hamiltonian. This Hamiltonian is composed of four terms that represent different physical behaviors of diblock polymers: the sum amount conservation term, the Flory–Huggins interaction energy term, the gradient energy term and the Ohta-Kawasaki long-distance interaction energy term. The strength of the impact on each of the four terms is controlled by the parameters ${\alpha }_{F}, {\alpha }_{I}, {\alpha }_{A}$ and ${\alpha }_{OK}$, respectively, and the parameter $f$ determines the fraction of monomer. The strength of the Ohta-Kawasaki energy term controls the fineness of the phase-separation structure. Note that Ising machines efficiently find the global minimum solution of a given Hamiltonian without becoming trapped at a local minimum solution. Thus, if a desired final state of a phase-field model is a local minimum free energy near the initial state, Ising machines may not be able to obtain the same results as the conventional method. Therefore, we report a new method of the phase-field model whose final simulation state must be the global minimum free energy.

To perform phase-field simulation by the Ising machine, we transform the phase-field model into a QUBO model. We set a two-dimensional calculation area and divide it into grids. We assign four binary variables to any *i*-th grid point ${S}_{i}$:

1 $${S}_{i}=\left\{{a}_{4i},{a}_{4i+1},{a}_{4i+2},{a}_{4i+3}\right\},$$

and we express the order parameter of the *i*-th grid point as ${\sum }_{0\le k<4}{a}_{4i+k}$. Since ${a}_{4i+k}\in \{\mathrm{0,1}\}$, the value range of ${S}_{i}$ is from 0 to 4 in this case. If we assign more binary variables to each grid, the resolution of the result is expected to increase, and as a result, the total number of required binary variables also increases.

**Figure 3:**
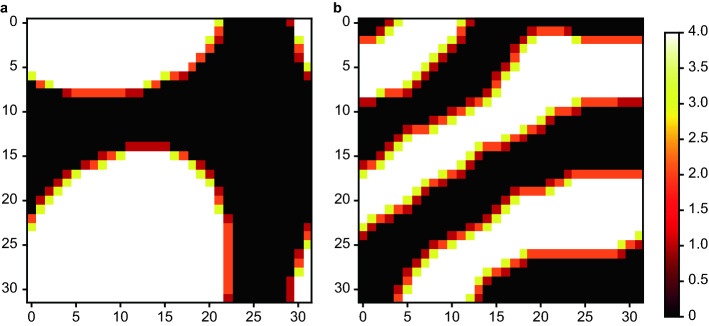
Results of a phase-field analysis in the case of $f=0.5$ at the equilibrium state for a quantum annealing simulator. (**a**) Without Ohta-Kawasaki energy and (**b**) with Ohta-Kawasaki energy. In (**b**), the observed microstructure corresponds to that in the phase diagram, i.e., a lamellar structure.

First, we conduct numerical simulation cases with and without Ohta-Kawasaki energy. We set the material properties in the analysis as follows: ${\alpha }_{I}=20.0, {\alpha }_{A}=5.0,{\alpha }_{OK}=0.0/0.4$ and $f=0.5$. Figure 3 shows the numerical analysis results of ${S}_{i}$. Clearly, the phases of polymer A and polymer B, shown in white and black, respectively, are separated into two large phases without Ohta-Kawasaki energy, as shown in Fig. 3a, while the composition of polymers A and B with Ohta-Kawasaki energy equilibrates with a finer pattern, as shown in Fig. 3b.

**Figure 4:**
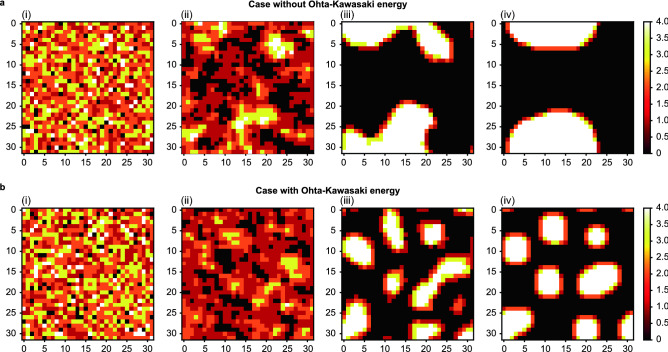
Temporal change until equilibrium is reached by the quantum annealing simulator in the case of $f=0.3$ (**a**) without Ohta-Kawasaki energy and (**b**) with Ohta-Kawasaki energy. As for the results obtained by the conventional phase-field analysis, the stronger the Ohta-Kawasaki energy becomes, the finer the microstructure at the equilibrium state. Without Ohta-Kawasaki energy, all the microstructures equilibrate as two large phases.

Figure 4 shows the emulation process of the annealing optimization in other cases with and without Ohta-Kawasaki energy. Note that this figure shows the results obtained when the Ising machine is interrupted before the process has finished and does not directly correspond to the physical time in the conventional method. We set ${\alpha }_{I}=20.0, {\alpha }_{A}=5.0,{ \alpha }_{OK}=0.0/0.5$ and $f=0.3.$ The simulation result without Ohta-Kawasaki energy (Fig. 4a(iv)) shows a larger pattern than the one with Ohta-Kawasaki energy (Fig. 4b(iv)). With the proposed Ising models, the simulation takes only 1 s.

**Figure 5:**
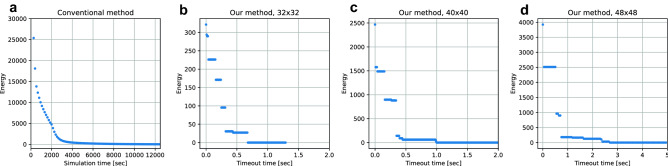
Energy during iterations of the conventional phase-field method and Ising machines. (**a**) Time evolution of the free energy in one simulation using the conventional method. (**b–d**) Minimum energy found in less than a specified time with different grid sizes.

To show the exact execution times of the two methods, we studied how the energy decreases with time in the conventional method and our new method in one simulation as an example. In Fig. 5a, we show the time evolution of the free energy of one randomly selected simulation using the conventional method. This figure shows that the simulation using the conventional method converges in about 4000 s (64 × 64 cells). Correspondingly, we performed some simulations using our new method with different timeout times. In Fig. 5b–d, we show the minimum energy that was found in less than a specified time. This figure shows that the simulations using Ising machines reach the minimum energy in about 0.8 s (32 × 32 cells), 1.5 s (40 × 40 cells), and 2.5 s (48 × 48 cells), which is very fast compared with the conventional method.

**Figure 6:**
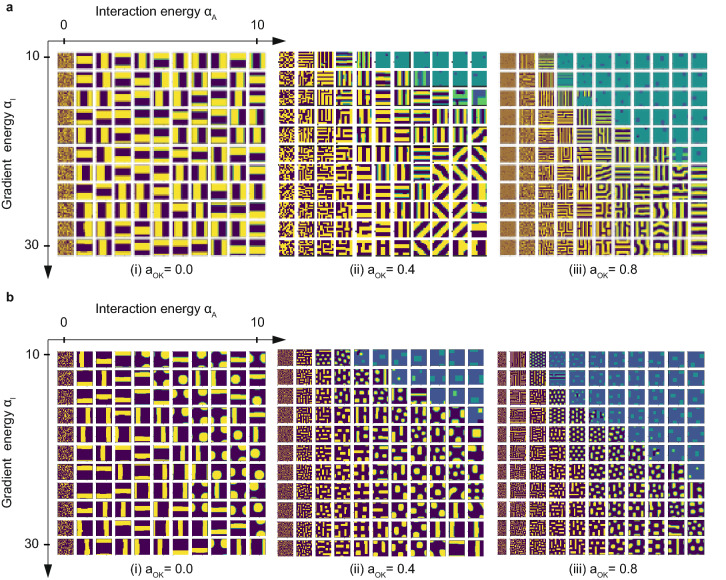
Large-scale parametric phase-field analyses for the quantum annealing simulator. (**a**) shows the case where the volumetric fraction is 0.5, and (**b**) shows the case where the volumetric fraction is 0.3. Using our proposed method, these simulations are completed in dozens of minutes. As for the previous simulation results, the microstructures are coincident with those in the phase diagram.

Finally, we carry out inclusively large-scale parametric analyses. Figure 6a shows the numerical analysis results in the case of fraction rate $f=0.5$, and Fig. 6b shows the case of $f=0.3$.

When you look at the phase diagram in Fig. 1, the case of $f=0.5$ shows only lamellar patterns, indicating that the parametric study using the defined Hamiltonian outputs physically correct results. In the case of $f=0.3$, hexagonal or cubic patterns should be observed from the phase diagram of Fig. 1, and it seems that the dotted pattern or the striped patterns may change depending on the viewing direction of these patterns because of the carrying out of two-dimensional calculations. Similar to the conventional phase-field analysis results, the characteristic length of the pattern seems to be shortened; thus, it makes sense that the pattern becomes finer as the long-distance interaction becomes stronger with increasing Ohta-Kawasaki energy.

When the coefficient of the gradient energy term becomes large, the parameter corresponding to the diffusion coefficient increases. This parameter change does not appear in the phase diagram in Fig. 1. The phase diagram should generally be created with the same material at a constant environmental temperature. However, the above outcome makes sense because the larger the gradient energy becomes, the larger the structure tends to be (the farther the material is distributed) in Fig. 6a,b. Additionally, in the region where the interaction energy is small, the pattern tends to be disordered, which is consistent with the phase diagram.

As shown in Fig. 6, the results are quite similar to those obtained by the conventional method, which means that the proposed modeling based on the Ising model is quite useful.

Note that there is no strict symmetry in the Ohta-Kawasaki term, but essentially, in the case of $f=0.7$ and $f=0.3$, the colors are simply swapped. If ${\alpha }_{OK}$ equals zero, there is strict symmetry; thus, the same pattern with completely inverted colors will appear. Additionally, since the Hamiltonian has symmetry when rotated 90 degrees about the *xy* axes, the direction of the lamellar structure appears randomly.

**Figure 7:**
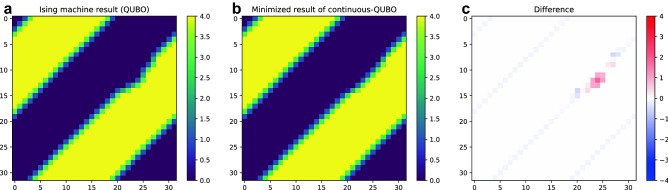
Difference between the result obtained with the Ising machine and the minimized result of continuous-QUBO. (**a**) One of the results obtained with the Ising machine, (**b**) corresponding optimal order parameters of continuous-QUBO, and (**c**) difference between the two results.

In terms of accuracy evaluation, the new QUBO model is not exactly the same as the conventional one and, thus, does not shared exactly the same parameters as the conventional method; making a direct comparison with the same model parameters is impossible. Instead of comparing the accuracy between the two methods, we evaluate errors due to the discretization of continuous order parameters in a simulation. In our QUBO model, the order parameters ${S}_{i}$ was discretized to (0, 1, 2, 3, 4). We now introduce a continuous version of our QUBO model, called continuous-QUBO, that is, the term ${\sum }_{k}{a}_{4i+k}$ in Eqs. (11)–(14) is replaced with continuous variables ${x}_{i}\in \left[\mathrm{0,4}\right]$. Then, the Hamiltonian of continuous-QUBO is minimized and the optimal order parameter ${x}_{i}^{*}$ is obtained using the gradient descent method. The error is calculated between the result obtained with the Ising machine, ${S}_{i}^{*}$, and the result of continuous-QUBO, ${x}_{i}^{*}$, which is considered the true value. This enables us to calculate the accuracy of the same model parameters. Figure 7 shows (a) one of the results obtained with the Ising machine, ${S}_{i}^{*}$, (b) the corresponding optimal order parameter of continuous-QUBO, ${x}_{i}^{*}$, and (c) the difference between the two plots. In this result, the mean error per cell ${\langle \left|{{S}_{i}^{*}-x}_{i}^{*}\right|\rangle }_{i}$ is about 0.018, which is sufficiently smaller than 1.0, which is the discretization size of the order parameter ${S}_{i}^{*}$.

In this study, we proposed a novel method to solve the phase-field model by the objective function for exploring the phase-separated structure of diblock polymers by an Ising machine, with an eye toward the use of quantum annealer. We confirmed the tendency from the obtained results and the performance by comparing them with the phase-field simulation. The approach involves the use of a global optimization metaheuristic algorithm, called simulated annealing, to directly minimize the Helmholtz free energy instead of minimizing it analytically and then solving the resulting nonlinear, partial differential equation, i.e., the governing equation of the phase-field model. As a result, we obtained the following:

1. The results obtained by Ising machine showed the same tendency as the results obtained from the conventional phase-field analysis.
2. The obtained results were consistent with the phase diagram.
3. The analysis time was shortened to the extent that high-speed comprehensive analysis becomes possible.

The proposed modeling based on the Ising model has been shown to be very useful. In the future, we will address the following remaining issues for the practical use of this method. First, we will investigate the usefulness of the intermediate results obtained with an Ising machine, which may correspond to the non-equilibrium time history of the distribution obtained by the conventional method. Second, as the number of bits that Ising machines can handle increases, it will be possible to apply our method to the 3D domain. Third, as noted above, a direct comparison with the same model parameters is not yet possible. We will clarify the relationship between the parameters of our model and these of the conventional model by introducing correction terms using machine learning methods.

This scheme on how to discretize the continuum variable and formulate the QUBO will help in solving the other problems related to continuum mechanics.

## Methods

### Theory of the phase-field model

The phase-field model for spinodal decomposition is introduced. First, the Cahn–Hilliard equation^31^ can be written as follows:

2 $$\frac{\partial c}{\partial t}=\nabla \cdot \left\{M\nabla \left(\frac{\delta F}{\delta c}\right)\right\},$$

where $c$ is the concentration of a phase, which is the order parameter in the phase-field model, $F$ is the free energy functional and $M$ is the mobility. Here, $F$ is formulated with consideration of the long-distance interaction as follows:

3 $$F={F}_{\mathrm{grad}}+{F}_{\mathrm{chem}}+{F}_{\mathrm{long}},$$

where ${F}_{\mathrm{grad}}$ is the gradient energy, ${F}_{\mathrm{chem}}$ is the chemical potential and ${F}_{\mathrm{long}}$ is the long-distance interaction. These variables are defined as

4 $${F}_{\mathrm{grad}}={\int }_{V}\left(\frac{{\kappa }_{a}}{2}{\left|\nabla {c}_{a}\right|}^{2}+\frac{{\kappa }_{b}}{2}{\left|\nabla {c}_{b}\right|}^{2}\right)dv,$$

5 $${F}_{\mathrm{chem}}=\chi {\int }_{V}\left(RT{c}_{a}\mathrm{ln}{c}_{a}+RT{c}_{b}\mathrm{ln}{c}_{b}\right)dv,$$

6 $${F}_{\mathrm{long}}=A{\int }_{V}{\Omega }_{ab}\left({\varvec{x}}-{{\varvec{x}}}^{^{\prime}}\right){c}_{a}\left({{\varvec{x}}}^{^{\prime}}\right){c}_{b}\left({\varvec{x}}\right)dv,$$

where the variables with indices $a$ and $b$ denote the quantities of phase $a$ and phase $b$, respectively. Additionally, $c$ is the concentration, $\kappa$ is the diffusion coefficient, and ${\varvec{x}}$ and ${{\varvec{x}}}^{^{\prime}}$ are the position vectors. Moreover, $\chi$ is the interaction coefficient (Flory), and $A$ is the coefficient of the long-distance interaction. These coefficients are defined by $\kappa =1/(12r(1-f))$ and $A={c}_{0}/(2{N}^{2}d{s}^{2}{f}^{2}(1-f{)}^{2})$, with the numerical constant ${c}_{0}$ defined as in a previous work^27^, the ratio of phase *b* denoted by $f$, the number of segments denoted by $N$ and the length of the segment denoted by $ds$. Here, we can see that ${c}_{0}$ is the constant associated with the long-distance interaction term derived from Ohta-Kawasaki energy. Note that ${c}_{a}+{c}_{b}=1$. The variation in the energy with respect to ${c}_{b}$ can be calculated as

7 $$\begin{aligned} \frac{\delta F}{{\delta c_{b} }} = & \frac{{\delta F_{{{\text{grad}}}} }}{{\delta c_{b} }} + \frac{{\delta F_{{{\text{chem}}}} }}{{\delta c_{b} }} + \frac{{\delta F_{{{\text{long}}}} }}{{\delta c_{b} }} \\ = & - \kappa_{b} \nabla^{2} c_{b} + \chi RT\ln c_{b} + A\mathop \smallint \limits_{{V^{\prime}}} \Omega_{ab} \left( {{\varvec{x}} - \user2{x^{\prime}}} \right)c_{a} \left( {\user2{x^{\prime}}} \right)dv^{\prime}, \\ \end{aligned}$$

Substituting Eq. (7) into the Cahn–Hilliard Eq. (2) and eliminating the index $b$, the equation can be reduced as

8 $$\begin{aligned} \frac{\partial c}{{\partial t}} = & \nabla \cdot \left[ {\kappa \nabla \left\{ { - a\nabla^{2} c + \chi RT\ln c + A\mathop \smallint \limits_{{V^{\prime}}} \Omega_{ab} \left( {{\varvec{x}} - \user2{x^{\prime}}} \right)c_{a} \left( {\user2{x^{\prime}}} \right)dv^{\prime}} \right\}} \right] \\ = & M\nabla^{2} \left[ {\mu + \mathop \smallint \limits_{{V^{\prime}}} \Omega_{ab} \left( {{\varvec{x}} - \user2{x^{\prime}}} \right)c_{a} \left( {\user2{x^{\prime}}} \right)dv^{\prime}} \right] \\ = & M\nabla^{2} \mu + M\mathop \smallint \limits_{{V^{\prime}}} \nabla^{2} \Omega_{ab} \left( {{\varvec{x}} - \user2{x^{\prime}}} \right)c_{a} \left( {\user2{x^{\prime}}} \right)dv^{\prime} \\ = & M\nabla^{2} \mu + M\mathop \smallint \limits_{{V^{\prime}}} \delta \left( {{\varvec{x}} - \user2{x^{\prime}}} \right)c_{a} \left( {\user2{x^{\prime}}} \right)dv^{\prime}. \\ \end{aligned}$$

Here, $\mu$ is defined as $\mu \equiv -\kappa {\nabla }^{2}c+\chi RT\mathrm{ln}c$, and $M$ is considered constant. For the implementation of Eq. (8) in a program code, the finite difference method is generally employed. In addition, $\delta ({\varvec{x}}-{{\varvec{x}}}^{^{\prime}})={\nabla }^{2}{\Omega }_{ab}({\varvec{x}}-{{\varvec{x}}}^{^{\prime}})$, and $\delta$ is the Green function, which is calculated through a Fourier transformation.

### Calculation by an Ising machine

We conducted the calculations using Fixstars Amplify Annealing Engine (Amplify AE)^32^ with timeout of 1 s. Amplify AE is GPU-based Ising machine that can handle 100,000 bit-class problems.

When solving a problem by using Ising machines, we must design QUBO models of the binary variables. The QUBO model is formulated as

9 $$H={\sum }_{i\le j}^{N}{Q}_{ij}{a}_{i}{a}_{j},$$

where $H$ is the Hamiltonian or the energy, $N$ is the number of binary variables, ${a}_{i}\in \{\mathrm{0,1}\}$ is a binary variable, and ${Q}_{ij}$ denotes the interaction parameters. Ising machines search the values $\{{a}_{i}\}$ so that the Hamiltonian $H$ is minimized. The diagonal and off-diagonal elements of $Q$ represent the strength of bias and quadratic interactions, respectively. Here, it is necessary to set ${Q}_{ij}$ properly depending on the problem.

### Hamiltonian

We formulate the Hamiltonian. We construct the whole Hamiltonian $H$ as a linear combination of the summation preservation term ${H}_{\text{sum}}$, the interaction (internal) energy term ${H}_{int}$, the gradient (adjacent) energy term ${H}_{\text{adj}}$, and the long-distance energy term (the so-called Ohta-Kawasaki energy) ${H}_{\text{long}}$:

10 $$H={H}_{\mathrm{sum}}+{H}_{\mathrm{int}}+{H}_{\mathrm{adj}}+{H}_{\mathrm{long}},$$

where

11 $${H}_{\mathrm{sum}}={\alpha }_{F}{\left({\sum }_{{S}_{i}}\left[{\sum }_{k}{a}_{4i+k}\right]-fN\right)}^{2},$$

12 $${H}_{\mathrm{int}}=-{\alpha }_{I}{\sum }_{{S}_{i}}{\left(\left[{\sum }_{k}{a}_{4i+k}\right]-2\right)}^{2},$$

13 $${H}_{\mathrm{adj}}={\alpha }_{A}{\sum }_{\left({S}_{i},{S}_{j}\right)\in {P}_{\mathrm{adj}}}{\left(\left[{\sum }_{k}{a}_{4i+k}\right]-\left[{\sum }_{k}{a}_{4j+k}\right]\right)}^{2},$$

14 $${H}_{\mathrm{long}}={\alpha }_{OK}{\sum }_{\left({S}_{i},{S}_{j}\right)\in {P}_{\mathrm{all}}}{g}_{ij}\left(\left[{\sum }_{k}{a}_{4i+k}\right]\left[{\sum }_{k}{a}_{4j+k}\right]\right).$$

The term ${H}_{\mathrm{sum}}$ constrains the total order parameter of space. Equation (11) uses the square constraint because a strict preservation term cannot be used in QUBO models. The constant ${\alpha }_{F}$ controls the strictness of preservation, and the constant $f$ denotes the target total ratio of the order parameter. The term ${H}_{\mathrm{int}}$ makes the order parameter of each point 0 or 4 to avoid intermediate states as much as possible, with strength ${\alpha }_{I}$. The term ${H}_{\mathrm{adj}}$ controls the strength of adjacent interactions by bringing closer the order parameter of adjacent pairs of the grid points together. ${P}_{\mathrm{adj}}$ denotes the set of all adjacent pairs. The coefficient ${\alpha }_{A}$ is the strength of adjacent interactions. The term ${H}_{\mathrm{long}}$ expresses the Ohta-Kawasaki energy, and ${g}_{ij}$ is defined as ${g}_{ij}\equiv 1/{r}_{ij}$, with the distance between grids *i* and *j* denoted by ${r}_{ij}$. ${P}_{\mathrm{all}}$ denotes the set of all pairs of all grid points.

Multiplying the coefficient of each term by a constant does not influence the simulation results; thus, we set ${\alpha }_{F}$ to 1. In this setting, the whole Hamiltonian contains at most a quadratic term of the variables $\left\{{a}_{i}\right\}$; therefore, rearranging the whole Hamiltonian immediately yields the coefficient of QUBO models $\left\{{Q}_{ij}\right\}$.

## Data Availability

The datasets used and analysed during the current study available from the corresponding author on reasonable request.
